# A newly isolated *Bacillus licheniformis* strain thermophilically produces 2,3-butanediol, a platform and fuel bio-chemical

**DOI:** 10.1186/1754-6834-6-123

**Published:** 2013-08-28

**Authors:** Lixiang Li, Lijie Zhang, Kun Li, Yu Wang, Chao Gao, Binbin Han, Cuiqing Ma, Ping Xu

**Affiliations:** 1 State Key Laboratory of Microbial Technology, Shandong University, Jinan 250100, People’s Republic of China; 2 State Key Laboratory of Microbial Metabolism, and School of Life Sciences & Biotechnology, Shanghai Jiao Tong University, Shanghai 200240, People’s Republic of China

**Keywords:** 2,3-butanediol, *Bacillus licheniformis*, Thermophilic fermentation, 2,3-butanediol dehydrogenase

## Abstract

**Background:**

2,3-Butanediol (2,3-BD), a platform and fuel bio-chemical, can be efficiently produced by *Klebsiella pneumonia*, *K. oxytoca*, and *Serratia marcescens*. However, these strains are opportunistic pathogens and not favorable for industrial application. Although some generally regarded as safe (GRAS) microorganisms have been isolated in recent years, there is still a demand for safe 2,3-BD producing strains with high productivity and yield under thermophilic fermentation.

**Results:**

*Bacillus licheniformis* strain 10-1-A was newly isolated for 2,3-BD production. The optimum temperature and medium pH were 50°C and pH 7.0 for 2,3-BD production by strain 10-1-A. The medium composition was optimized through Plackett–Burman design and response surface methodology techniques. With a two-stage agitation speed control strategy, 115.7 g/L of 2,3-BD was obtained from glucose by fed-batch fermentation in a 5-L bioreactor with a high productivity (2.4 g/L·h) and yield (94% of its theoretical value). The 2,3-BD produced by strain 10-1-A comprises (2*R*,3*R*)-2,3-BD and *meso*-2,3-BD with a ratio of nearly 1:1. The *bdh* and *gdh* genes encoding *meso*-2,3-butanediol dehydrogenase (*meso*-BDH) and glycerol dehydrogenase (GDH) of strain 10-1-A were expressed in *Escherichia coli* and the proteins were purified. *meso*-2,3-BD and (2*R*,3*R*)-2,3-BD were transformed from racemic acetoin by *meso*-BDH and GDH with NADH, respectively.

**Conclusions:**

Compared with the reported GRAS 2,3-BD producers, *B. licheniformis* 10-1-A could thermophilically produce 2,3-BD with a high concentration, productivity and yield. Thus, the newly isolated GRAS strain 10-1-A might be a promising strain for industrial production of 2,3-BD. Two key enzymes for *meso*-2,3-BD and (2*R*,3*R*)-2,3-BD production were purified and further studied, and this might be helpful to understand the mechanism for 2,3-BD stereoisomers forming in *B. licheniformis*.

## Background

2,3-Butanediol (2,3-BD) is a crucial vicinal diol with 3 stereoisomers: *meso*-2,3-BD, (2*R*,3*R*)-2,3-BD, and (2*S*,3*S*)-2,3-BD. As an important commodity chemical, it can be used as the starting material for some bulk chemicals such as methyl ethyl ketone, gamma-butyrolactone and 1,3-butadiene [1-3]. It also has potential applications in the manufacture of food additives, cosmetics, printing ink, perfumes, drugs and explosives [4]. With a heating value of 27,200 J/g, 2,3-BD compares favourably with ethanol (29,100 J/g) and methanol (22,100 J/g) for using as a liquid fuel or fuel additive [4]. Enantio pure 2,3-BD, such as (2*R,*3*R*)-2,3-BD, can be used as an antifreeze agent [5]. In addition, *meso*-2,3-BD can be used for the synthesis of renewable polyesters [6]. Nowadays, it is considered that the key downstream products of 2,3-BD have a potential global market of around 32 million tonnes per annum [1].

2,3-BD can be produced via chemical or biotechnological routes [2,3]. Due to the gradual exhaustion of crude oil reserves, interest in the biotechnological production of 2,3-BD has increased remarkably in recent years. Currently, *Klebsiella pneumonia*, *K. oxytoca* and *Serratia marcescens* are regarded as the most efficient 2,3-BD production strains that mainly produce *meso*-2,3-BD [7-9]. However, these strains are all categorized by the World Health Organization (WHO) as risk group 2 species (pathogenic) [4] and thus not desirable in industrial-scale production of 2,3-BD [1].

In recent years, some generally regarded as safe (GRAS) organisms including *Bacillus licheniformis*, *Paenibacillus polymyxa* (formerly *B. polymyxa*), and *B. amyloliquefaciens* have been isolated for 2,3-BD production [10-12]. *Saccharomyces cerevisiae*, *B. licheniformis* and *B. subtilis* have also been metabolically engineered to produce 2,3-BD [13-15]. The maximum 2,3-BD concentration obtained by a GRAS strain was 144.0 g/L, which was comparable with those achieved by the risk group 2 strains [10]. However, these reported GRAS strains for 2,3-BD production have optimal fermentation temperature of 30-40°C. Due to the moderate temperature conditions, 2,3-BD fermentations can be easily contaminated [16]. Thermophilic fermentation, typically operated at 50-60°C, can reduce the risk of bacterial contamination [17]. In addition, thermophilic fermentation is more efficient for utilization of lignocelluloses by simultaneous saccharification process [15]. Recently, Xiao et al. isolated a thermophilic strain, *Geobacillus* sp. XT15, for 2,3-BD and acetoin (AC) production [17]. However, the concentration, productivity and yield of the 2,3-BD produced under thermophilic fermentation were rather low.

2,3-BD was produced from AC, an intermediate of 2,3-BD fermentation in microbes. Thus, 2,3-butanediol dehydrogenase (BDH), which catalyzes the interconversion between AC and 2,3-BD, is a key enzyme for 2,3-BD production. Several BDHs with different stereospecificities have been purified and characterized in previous studies [18-24]. The *meso*-BDHs in *K. pneumoniae*, *S. marcescens* and *Enterobacter cloacae* belong to the short-chain dehydrogenase/reductase (SDR) family. In the presence of NADH, all of three *meso*-BDHs could catalyze the conversion of (3*R*)-AC to *meso*-2,3-BD and (3*S*)-AC to (2*S*,3*S*)-2,3-BD [18,21,23]. The (2*R*,3*R*)-BDHs from *P. polymyxa*, *B. subtilis*, and *S. cerevisiae* belong to the medium-chain dehydrogenase/reductase family (MDR) [18]. These enzymes exhibited the abilities in interconversion of (3*S*)-AC/*meso*-2,3-BD and (3*R*)-AC/(2*R*,3*R*)-2,3-BD [19,20,24]. On the other hand, it was reported that glycerol dehydrogenase (GDH) in *Ogataea angusta* could catalyze interconversion between (3*R*)-AC and (2*R*,3*R*)-2,3-BD [25]. It might be another enzyme for 2,3-BD production.

In this work, a thermophilic GRAS strain *B. licheniformis* 10-1-A was newly isolated for 2,3-BD production. After statistical optimization of the fermentation medium composition and the culture conditions, 2,3-BD production was carried out in fed-batch fermentation by using two-stage agitation speed control strategy. In addition, the enzymes related to production of (2*R*,3*R*)-2,3-BD and *meso*-2,3-BD in strain 10-1-A were also investigated.

## Results

### Isolation of thermophilic bacteria for 2,3-BD production

To screen a thermophilic strain for the production of 2,3-BD, about 63 isolates were obtained from 10 soil samples with deMan-Rogosa-Sharpe (MRS) medium at 50°C. Strain 10-1-A that produced the highest concentration of 2,3-BD was selected for further study.

Cells of the strain are a rod shaped, with cell diameters around 0.6-0.8 μm. The strain is Gram positive, starch hydrolysis positive, facultative growth positive, Voges-Proskauer (VP) reaction positive, and citrate utilization positive. The strain could grow in 6.5% NaCl and at 55°C. The partial 16S rDNA sequence of strain 10-1-A (GenBank: JQ965662) is 100% identical with that of *B. licheniformis* strain CICC 10101 (GenBank: GQ375234). Based on the above results, strain 10-1-A was identified as a strain of *B. licheniformis*. This strain was deposited at the China General Microbiological Culture Collection Center (CGMCC 5461).

### Optimization of fermentation temperature

**Figure 1:**
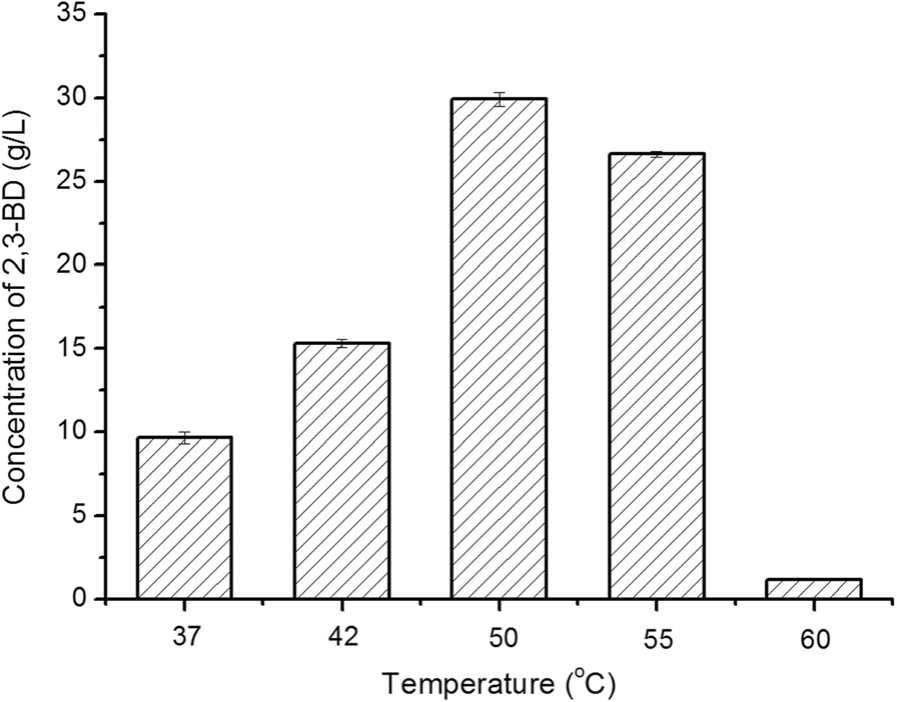
Effect of temperature on 2,3-BD production by strain 10-1-A.

The effect of temperature on the production of 2,3-BD was studied. Experiments were performed in the MRS medium at 37°C, 42°C, 50°C, 55°C, and 60°C, respectively. As shown in Figure 1, the highest concentration of 2,3-BD, 29.9 ± 0.4 g/L, was obtained at 50°C. This temperature was then selected for future investigations.

### Optimization of initial glucose concentration

**Figure 2:**
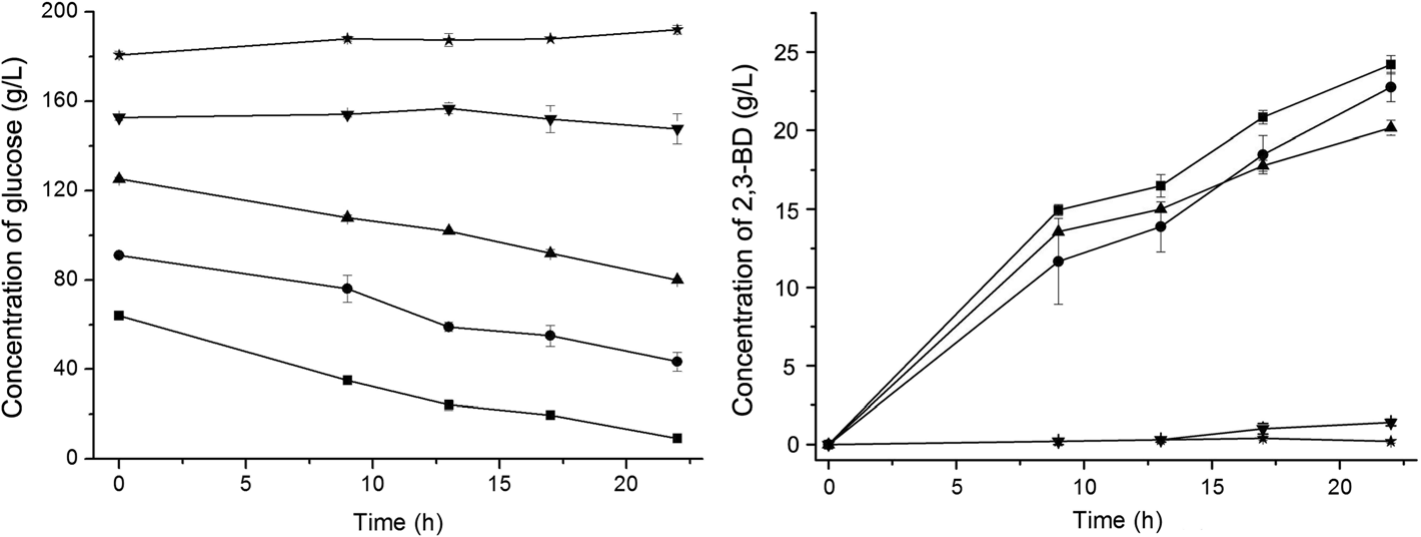
**Effect of initial glucose concentration on 2,3-BD production by strain 10-1-A.** The initial glucose concentrations used were at 64.0 g/L (■), 91.0 g/L (●), 125.0 g/L (▲), 152.0 g/L (▼) and 180.0 g/L (★). The experiments were conducted in 500-mL Erlenmeyer flasks containing 100 mL of each medium with shaking at 180 rpm on a rotary shaker at 50°C.

To select the initial carbon source concentration, the effect of glucose concentration on 2,3-BD production was investigated at initial pH 6.5, 50°C, and 180 rpm. As shown in Figure 2, with initial glucose concentrations lower than 125.0 g/L, similar concentrations of 2,3-BD were acquired. When the initial glucose concentration higher than 152.0 g/L, utilization of glucose was obviously inhibited and a small amount of 2,3-BD was produced. Initial glucose concentration between 64.0 g/L and 125.0 g/L was thus utilized in the successive studies.

### Selection of the organic nitrogen source

**Figure 3:**
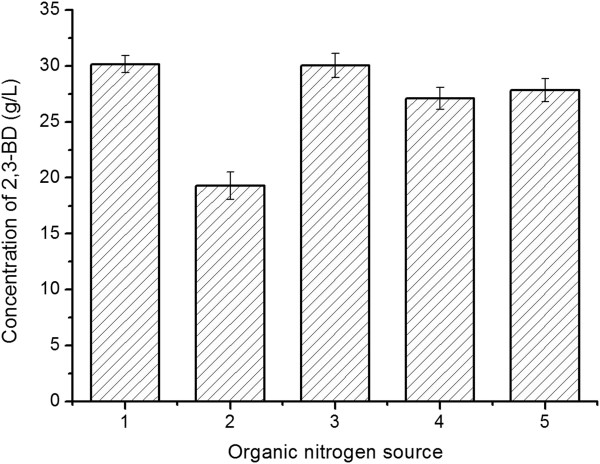
**Effect of organic nitrogen source on 2,3-BD production.** 1, 0.5% YE, 1% peptone and 1% beef extract; 2, 1% CSLP; 3, 1% CSLP and 0.5% YE; 4, 1% CSLP and 0.5% peptone; 5, 1% CSLP and 0.5% beef extract. The experiments were conducted in 500-mL Erlenmeyer flasks containing 100 mL of each medium with shaking at 180 rpm on a rotary shaker at 50°C.

Corn steep liquor powder (CSLP), an inexpensive organic nitrogen source available on a large scale, was used to replace the expensive organic nutrient in the selected medium. However, when yeast extract (YE), peptone, and beef extract in the fermentation medium were completely replaced with 10.0 g/L CSLP, the concentration of 2,3-BD would decreased from 30.2 ± 0.8 g/L to 19.3 ± 1.2 g/L (Figure 3). To obtain high concentration of 2,3-BD by using cheap CSLP as the major organic nitrogen source, 5.0 g/L of YE, peptone or beef extract was added in the fermentation medium containing 10.0 g/L of CSLP. The concentration of 2,3-BD in the medium containing 5.0 g/L YE and 10.0 g/L CSLP was higher than others. Thus, 5.0 g/L YE and 10.0 g/L CSLP were chosen as the organic nitrogen source of the fermentation medium for future investigations.

### Statistical optimization of fermentation medium

Phosphate, acetate, ammonium salt, Mg^2+^, YE and CSLP were used to compose the Plackett–Burman design (Table 1 and Table 2). Effects of the variables on the response and the significant levels are shown in Table 1.

**Table 1:** Plackett–Burman design for screening the variables, effect of each variable on 2,3-BD production, and statistical analysis of variables in the fermentation of glucose using strain 10-1-A

**Factor**	**Variable**	**Low level**	**High level**	**Coefficient**	**Standard error**	***T*-value**	***P*-value**
YE	*X*_1_	2	4	2.061	0.5149	4	0.01 ^a^
CSLP	*X*_2_	5	10	2.335	0.5149	4.53	0.006 ^a^
Sodium acetate	*X*_3_	2	4	2.321	0.5149	4.51	0.006 ^a^
K_2_HPO_4_·3H_2_O	*X*_4_	2	4	1.051	0.5149	2.04	0.097
Triammonium citrate	*X*_5_	1	2	−1.160	0.5149	−2.25	0.074
MgSO_4_·7H_2_O	*X*_6_	0.25	0.5	−0.567	0.5149	−1.1	0.321

R^2^ = 0.9309, R^2^ (Adj) = 0.8480.

^a^ Statistically significant at 95% of confidence level (*p* < 0.05).

**Table 2:** The level code for each variable in the Plackett–Burman design and corresponding production response of 2,3-BD

**Run**	**Variable levels**						**2,3-BD**
	***X*_1_**	***X*_2_**	***X*_3_**	***X*_4_**	***X*_5_**	***X*_6_**	**(g/L)**
1	1	−1	1	−1	−1	−1	30.47 ± 0.06
2	1	1	−1	1	−1	−1	32.16 ± 0.64
3	−1	1	1	−1	1	−1	28.63 ± 0.01
4	1	−1	1	1	−1	1	31.31 ± 0.67
5	1	1	−1	1	1	−1	29.55 ± 0.68
6	1	1	1	−1	1	1	29.55 ± 0.28
7	−1	1	1	1	−1	1	30.28 ± 0.61
8	−1	−1	1	1	1	−1	26.67 ± 0.97
9	−1	−1	−1	1	1	1	19.31 ± 0.65
10	1	−1	−1	−1	1	1	22.31 ± 0.49
11	−1	1	−1	−1	−1	1	26.82 ± 0.64
12	−1	−1	−1	−1	−1	−1	18.90 ± 0.59

To approach the neighborhood of the optimum response, a fitted first-order model was obtained from the Plackett–Burman experimental design as follows:

(1) $$\begin{array}{ll} \;Y= & \;27.16+2.06X_{1}+2.34X_{2}+2.32X_{3}\\ & \;+1.05X_{4}–1.16X_{5}–0.57X_{6} \end{array}$$

The coefficient of each variable represents its effect extent on the production of 2,3-BD. The linear regression coefficient (R^2^) is 0.9309 and the adjusted determination coefficient (Adj R^2^) is 0.8480 for the model, indicating that the model would be reasonable for the Plackett–Burman design.

Based on the statistical analysis, the factors having the greatest impacts on the production of 2,3-BD were identified as *X*_1_ (YE, *p* = 0.010), *X*_2_ (CSLP, *p* = 0.006) and *X*_3_ (sodium acetate, *p* = 0.0050). *X*_4_ (K_2_HPO_4_·3H_2_O) was set at its high level according to its positive effect. *X*_5_ (Triammonium citrate) and *X*_6_ (MgSO_4_·7H_2_O) were set at their low levels according to their negative effects.

According to the above first-order equation, the steepest ascent direction is proportional to (2.061, 2.335, 2.321), approximately equivalent to (0.9, 1.0, 1.0). The design and responses of the steepest ascent experiment are shown in Table 3. The concentration of 2,3-BD was highest when the concentration of YE, CSLP and sodium acetate were selected as 5.7, 15.0 and 6.0 g/L, respectively. This combination was used as an appropriate center point for the central composition design (CCD) experiment. The design matrix of the variables together with the experimental responses is shown in Table 4.

**Table 3:** Experimental design and results of the steepest ascent

**Run**	**YE (g/L)**	**CSLP (g/L)**	**Sodium acetate (g/L)**	**2,3-BD (g/L)**
1	3	7.5	3	28.6
2	3.9	10	4	32.6
3	4.8	12.5	5	39.1
4	5.7	15	6	41.3
5	6.6	17.5	7	38.9

**Table 4:** Experimental design and results of central composition experiment

**Run**	**Coded variable level**			**2,3-BD (g/L)**	
	***X*_**1**_**	***X*_**2**_**	***X*_**3**_**	**Observed**	**Predicted**
1	−1	−1	−1	32.2	32.2
2	1	−1	−1	35.0	35.0
3	−1	1	−1	34.0	34.9
4	1	1	−1	33.6	32.6
5	−1	−1	1	35.7	35.8
6	1	−1	1	41.0	39.2
7	−1	1	1	37.9	36.9
8	1	1	1	36.1	35.3
9	−1.682	0	0	36.2	35.8
10	1.682	0	0	35.2	36.8
11	0	−1.682	0	33.8	34.4
12	0	1.682	0	32.7	33.3
13	0	0	−1.682	34.5	34.1
14	0	0	1.682	37.7	39.3
15	0	0	0	41.3	41.8
16	0	0	0	43.0	41.8
17	0	0	0	40.7	41.8
18	0	0	0	42.7	41.8
19	0	0	0	41.6	41.8
20	0	0	0	41.8	41.8

The *p*-values were used as a tool to check the significance of each coefficient (Table 5). The smaller the *p* value, the more significant is the corresponding coefficient [26]. Model terms are significant for values of *p* less than 0.05. According to these results, *X*_3_ (sodium acetate), *X*_1_^2^ (YE), *X*_1_*X*_2_, *X*_2_^2^ (CSLP) and *X*_3_^2^ are significant model terms.

**Table 5:** Significance test of regression coefficient of the central composition experiment

**Variable**	**Coefficient**	**Standard error**	***T*-value**	***P*-value**
Intercept	41.8017	0.5181	80.6810	0.0002
*X*_1_	0.3089	−0.3440	0.8979	0.3903
*X*_2_	−0.3078	−0.3440	−0.8946	0.3920
*X*_3_	1.5652	−0.3440	4.5496	0.0010 ^a^
*X*_1_^2^	−1.9426	−0.3440	−5.8005	0.0002 ^a^
*X*_1_*X*_2_	−1.2662	0.4495	−2.8170	0.0182 ^a^
*X*_1_*X*_3_	0.1538	0.4495	0.3420	0.7394
*X*_2_^2^	−2.8194	0.3349	−8.4185	0.0001 ^a^
*X*_2_*X*_3_	−0.3888	0.4495	−0.8649	0.4074
*X*_3_^2^	−1.8012	0.3349	−5.3782	0.0003 ^a^

R^2^ = 0.9350, R^2^ (Adj) = 0.8766.

^a^ Statistically significant at 95% of confidence level (*p* < 0.05).

By applying multiple regression analysis to the experimental data, the following second-order polynomial equation was found:

(2) $$\begin{array}{ll} \;Y=\; & \;41.80+0.31X_{1}–0.31X_{2}\\ & \;+1.56X_{3}–1.94X_{1}{}^{2}–2.82X_{2}{}^{2}–1.80X_{3}{}^{2}–1.26X_{1}X_{2}\\ & \;+0.15X_{1}X_{3}–0.39X_{2}X_{3} \end{array}$$

Where *Y* is the predicted 2,3-BD production (g/L); *X*_1_, *X*_2_ and *X*_3_ are the coded values of YE, CSLP and sodium acetate, respectively. The quality of fit of the quadratic regression model equation is expressed by the coefficient of determination (R^2^), which equals 0.9350, indicating that 93.5% of the variability in the response could be explained by the model. The value of Adj R^2^ (0.8766) is also very high to advocate for a high significance of the model. These results indicated that the response equation provides a suitable model for the CCD experiment.

**Figure 4:**
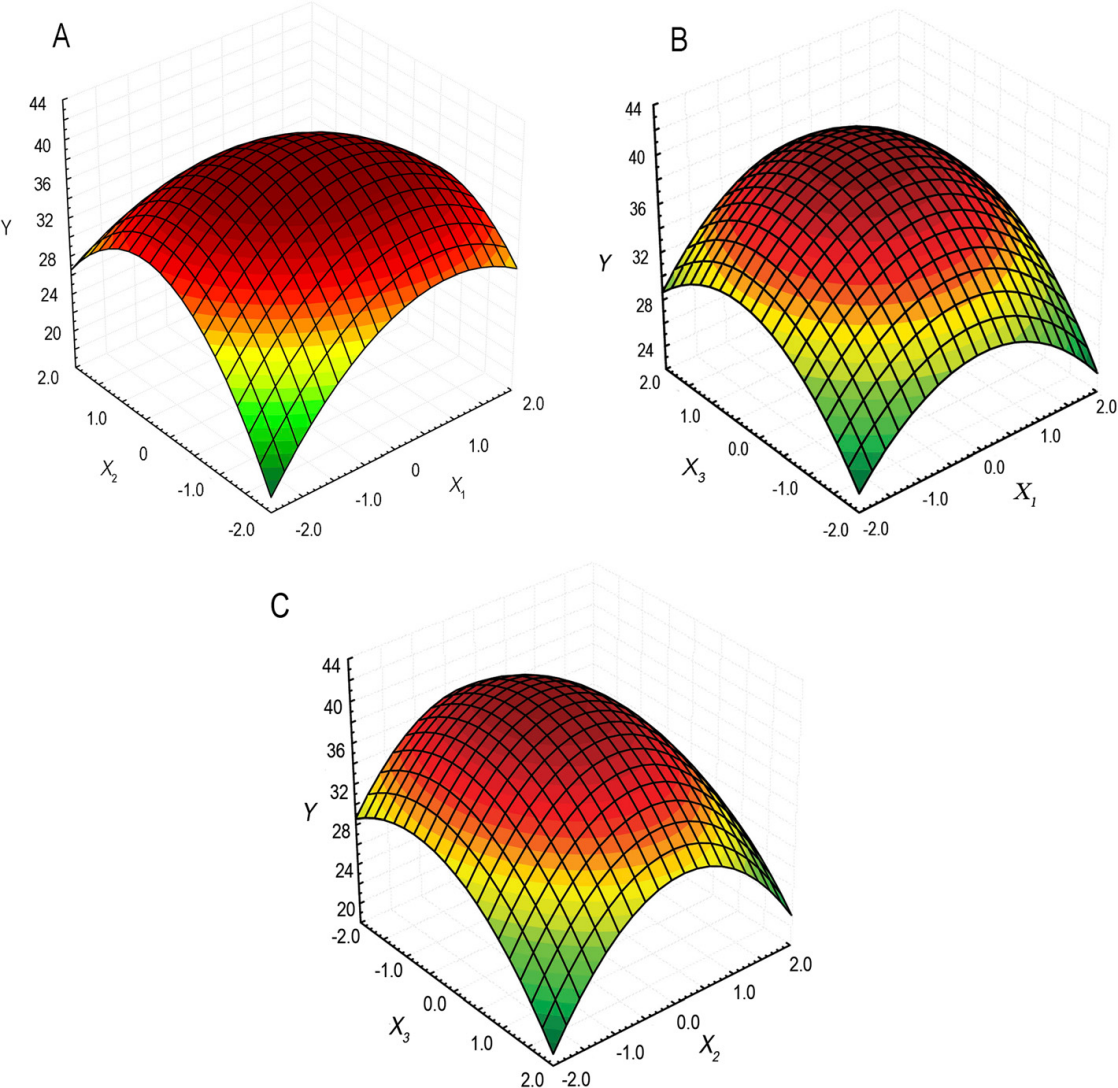
**The response surface plot. A**, effects of YE and CSLP on 2,3-BD production. **B**, effects of YE and sodium acetate on 2,3-BD production. **C**, effects of CSLP and sodium acetate on 2,3-BD production.

The three-dimensional graph for the response surface model is shown in Figure 4. It is evident from the plot that 2,3-BD production has a maximum point in the studied region. When *X*_1_ = 0.1353 (YE, 5.8 g/L), *X*_2_ = −0.1162 (CSLP, 14.7 g/L) and *X*_3_ = 0.4528 (sodium acetate, 6.5 g/L), 2,3-BD concentration would reach the predicted maximum point (42.2 g/L).

In order to validate the adequacy of the model, the confirmation experiment was carried out at the predicted optimal condition. The mean concentration of the obtained 2,3-BD from triplicate trials in shaking flask was 42.3 ± 1.4 g/L, which is near the predicted value (42.2 g/L).

### Effect of pH on 2,3-BD production

The effects of pH (6.5-8.0) of the culture medium on glucose utilization and 2,3-BD production were investigated. The experiments were carried out with 105.0 g/L glucose for 11 h in a 1-L bioreactor with 0.8 L initial medium. The results are summarized in Table 6. 2,3-BD production by *B. licheniformis* 10-1-A is obviously pH-dependent with the maximum productivity at pH 7.0. The yield of 2,3-BD under the low pH is above 100% of the theoretical yield, which might be caused by the addition of acetic acid in the fermentation process. Thus, pH 7.0 was chosen for further experiments.

**Table 6:** **Effect of pH on 2,3-BD production by *B. licheniformis *10-1-A**

**pH**	**Glucose consumed**	**Products (g/L)**						**Yield**	**Productivity**
								**(%)**	**(g/L·h)**
	**(g/L)**	**2,3-BD**	**AC**	**Lactic acid**	**Formic acid**	**Acetic acid**	**Ethanol**		
6.5	74.0	36.6	0.6	1.2	1.5	5.5	4.1	100.7	3.3
7.0	99.0	49.6	1.8	1.3	1.3	4.9	8.3	100.4	4.6
7.5	92.0	41.2	0.8	8.1	15.2	1.2	3.6	91.4	3.8
8.0	43.0	4.5	0.1	15.5	1.5	4.0	1.9	21.2	0.4

### Two-stage agitation speed control strategy for 2,3-BD fermentation

**Figure 5:**
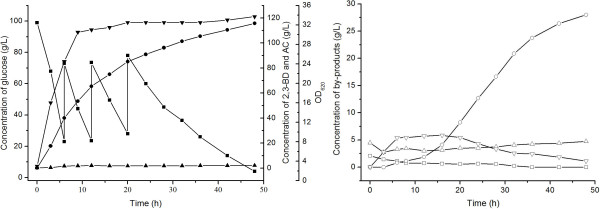
**Time course of fed-batch fermentation of 2,3-BD.** Biomass (▼), 2,3-BD (●), glucose (■), AC (▲), acetic acid (Δ), lactic acid (□), ethanol (▽) and formic acid (○). The fermentation was carried out at 50°C in a 5-L bioreactor with pH control by automatic addition of 6 M acetic acid and NaOH, stirring at 400 rpm in the first 10 h and then decreased to 200 rpm, and airflow at 1.0 vvm.

Fed-batch fermentation was performed in a 5-L bioreactor by using the optimal medium at pH 7.0, and different agitation speed control strategies were used. When the agitation speed was 400 rpm, the AC increased quickly in the stabilization phase that lead to a low yield of 2,3-BD (Table 7). Meanwhile, when the agitation speed was 200 rpm, formic acid increased quickly after 2 h to a high concentration of 42.0 g/L at the end of fermentation (Table 7). This also decreased the yield of 2,3-BD. To overcome these problems, a two-stage agitation speed control strategy for 2,3-BD fermentation was used. In first 10 h, the agitation speed was 400 rpm, and then it was decreased to 200 rpm. As shown in Figure 5, the feeding started at 6 h when the residual glucose concentration was below 20.0 g/L. The curve of 2,3-BD production can be divided into two parts (Figure 5). In the first 20 h, 85.0 g/L of 2,3-BD was produced and the average productivity reached 4.3 g/L·h. From 20 h to 48 h, the concentration of 2,3-BD climbed from 85.0 g/L to 115.7 g/L and the average productivity was 1.1 g/L·h. At the end of the fed-batch fermentation, 115.7 g/L of 2,3-BD was obtained from 246.0 g/L glucose after 48 h of fermentation at 50°C. The yield of 2,3-BD produced by *B. licheniformis* 10-1-A was 94% of the theoretical value and the average productivity of 2,3-BD was 2.4 g/L·h. Formic acid was the main by-product and its concentration was 28.3 g/L at 48 h. The time course of other by-products was shown in Figure 5. Lactic acid (2.0 g/L) was depleted in the process. The acetic acid was used as the neutralizer in the fermentation process while the concentration of acetic acid was maintained at about 4.0 g/L.

**Table 7:** 2,3-BD fermentation by strain 10-1-A with different agitation speed control strategies

**Control strategy**	**Glucose consumed**	**Products (g/L)**						**Yield**	**Productivity**
								**(%)**	**(g/L·h )**
	**(g/L)**	**2,3-BD**	**AC**	**Lactic acid**	**Formic acid**	**Acetic acid**	**Ethanol**		
A	189	81.0	0.9	0.8	42	2.2	1.8	85.7	2.3
B	185	45.7	45.1	0	1.2	3.3	0.6	49.4	1.6
C	246	115.7	2.2	0	28.3	4.8	1.1	94.1	2.4

A: the agitation speed was 200 rpm in the course; B: the agitation speed was 400 rpm in the course; C: the agitation speed was 400 rpm in the first 10 h and then decreased to 200 rpm.

### Stereoisomeric composition analysis of 2,3-BD produced by strain 10-1-a

**Figure 6:**
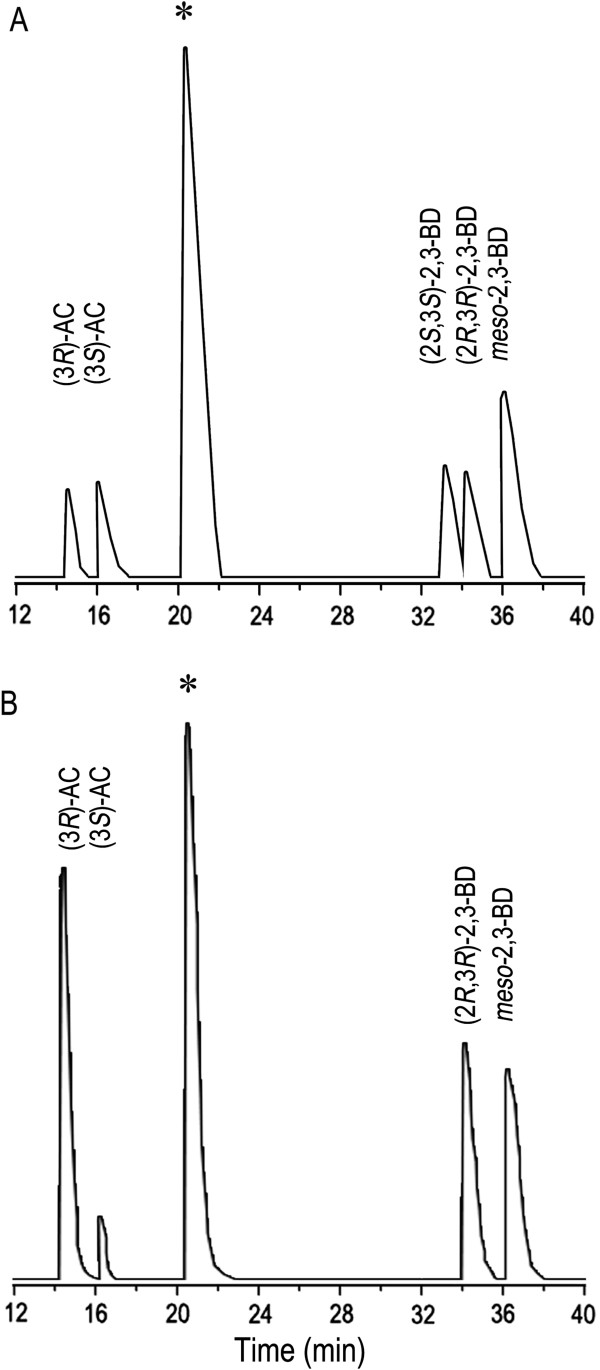
**Analysis of the 2,3-BD stereoisomers produced by strain 10-1-A. A**, GC analysis of the standard chemicals. **B**, GC analysis of the 2,3-BD produced by strain 10-1-A in 500-mL Erlenmeyer flasks experiment. * Isoamyl alcohol was used as the internal standard.

The stereoisomeric composition of 2,3-BD formed by bacteria differs among strains. The 2,3-BD produced by strain 10-1-A was analyzed by GC with a flame ionization detector and a fused silica capillary column [24]. It was found that there was a mixture of (2*R*,3*R*)-2,3-BD and *meso*-2,3-BD with a ratio of nearly 1:1 in the broth as shown in Figure 6.

Genome sequence of strain 10-1-A contains a complete *alsSD* operon for (3*R*)-AC production [27]. Since (2*R*,3*R*)-2,3-BD and *meso*-2,3-BD could be produced from (3*R*)-AC by (2*R*,3*R*)-BDH and *meso*-BDH, respectively, the putative (2*R*,3*R*)-BDH and *meso*-BDH encoding genes in strain 10-1-A were analyzed based on multiple sequence alignment and phylogenetic tree analysis (Additional file 1: Figure S1).

**Figure 7:**
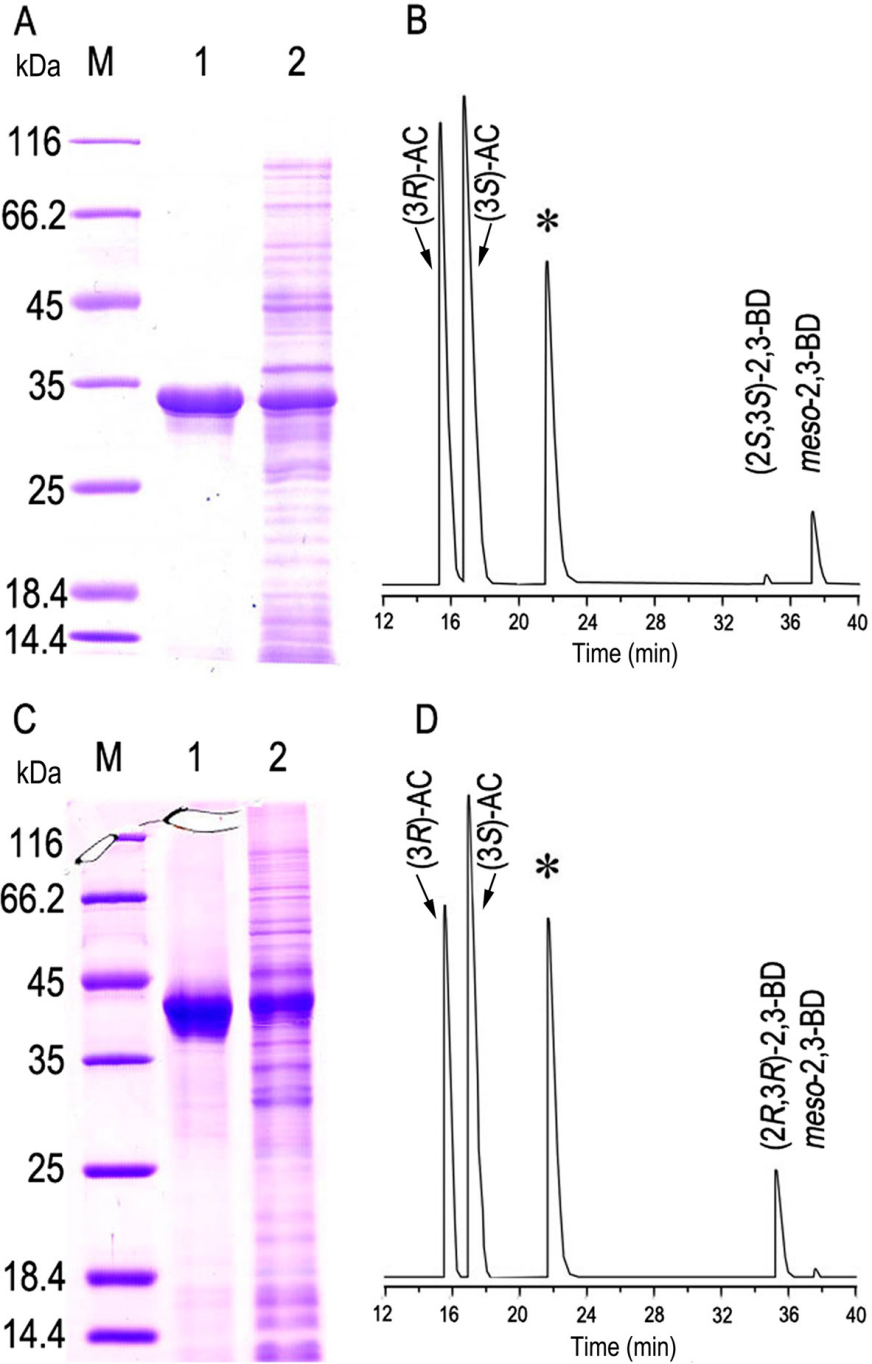
**SDS-PAGE of the purified enzymes and GC analysis of the reaction products from AC. A**, SDS-PAGE analysis of the purified *meso*-BDH: M, marker; line 1, purified *meso*-BDH; line 2, crude extract of *E. coli* BL21 (DE3) (pETDuet-*bdh*) induced by IPTG. **B**, GC analysis of the *meso*-BDH catalyzed products of AC with NADH as a cofactor. **C**, SDS-PAGE analysis of the purified GDH: M, marker; line 1, purified GDH; line 2, crude extract of *E. coli* BL21 (DE3) (pETDuet-*gdh*) induced by IPTG. **D**, GC analysis of the GDH catalyzed products of AC with NADH as a cofactor. * Isoamyl alcohol was used as the internal standard in **B** and **D**.

The enzymes encoding by *bdh* (GenBank: KF250429) and *gdh* (GenBank: KF250430) were suspected to be able to catalyze AC to 2,3-BD and these genes were cloned and expressed in *Escherichia coli* (BL21). Both of the enzymes were purified and detected by sodium dodecyl sulfate polyacrylamide gel electrophoresis (SDS-PAGE) as shown in (Figure 7A and C). When 100 mM AC was incubated with 100 mM NADH and 10 U purified *meso*-BDH for 30 min in 50 mM Tris–HCl buffer (pH 7.4), *meso*-2,3-BD and a little (2*S*,3*S*)-2,3-BD were produced (Figure 7B). While the purified GDH was used under the same conditions as assaying *meso*-BDH, (2*R*,3*R*)-2,3-BD and a little *meso*-2,3-BD were produced (Figure 7D).

## Discussion

Thermophilic fermentation can reduce the risk of bacterial contamination, and can even be operated without sterilization [28], making it more efficient and cost-effective. In addition, thermophilic fermentation matches the simultaneous saccharification process for utilization of lignocelluloses, which has the best efficiency around 50°C [15]. Thus, isolation of thermophilic strains for lactic acid, ethanol and 2,3-BD production has gained considerable attention [15,17,28-30]. Wang et al. reported the first high temperature fermentation of 2,3-BD by using a genetically engineered *B. licheniformis* strain BL5 in which the *ldh* gene was knocked out [15]. The concentration of 2,3-BD produced by *B. licheniformis* BL5 was 12.1 g/L. Xiao et al. isolated a thermophilic *Geobacillus* strain XT15 and found it is an AC and 2,3-BD producer. The concentration of 2,3-BD produced by strain XT15 was 14.5 g/L [17]. In this work, a *B. licheniformis* strain 10-1-A was isolated for 2,3-BD production. In MRS medium, the concentration of 2,3-BD obtained by strain 10-1-A at 50°C was about 30.0 g/L. Strain 10-1-A might be a promising thermophilic strain and thus the conditions for 2,3-BD production by the strain were optimized.

Statistically based experimental designs were used to find an appropriate fermentation medium for strain 10-1-A. During the optimization process, CSLP, an inexpensive valuable nutrient source available on a large scale, was used as the major nitrogen source to replace the expensive nutrient source. The 2,3-BD concentration in the optimized medium is 1.4-fold than that of MRS medium. Then, the operating conditions for 2,3-BD production from glucose by strain 10-1-A was also optimized. It was reported that pH 6.0 was the optimal condition for production of 2,3-BD by *B. licheniformis*[31]. For *B. licheniformis* BL5, pH 5.0 was regarded as the optimum pH for 2,3-BD production [15]. The results in this study shows that pH was also a major influence on 2,3-BD production and pH 7.0 was optimum for strain 10-1-A. Oxygen supply is another important variable in the 2,3-BD fermentation which could affect product yield, productivity, and by-products formation [3,4]. Agitation speed control was found to be a simple method to adjust oxygen supply and realize efficient 2,3-BD fermentation [7]. In this work, a two-stage agitation speed control strategy was found to be more suitable for 2,3-BD fermentation by strain 10-1-A (Table 7). Under optimum conditions, 115.7 g/L of 2,3-BD was obtained from 246.0 g/L glucose after 48 h of fermentation by using the novel strain 10-1-A.

Due to their GRAS status, the 2,3-BD produced by *B. licheniformis*, *P. polymyxa*, and *B. amyloliquefaciens* might afford the demand for the production of food additives or cosmetics (Table 8). Although *P. polymyxa* DSM365 produced 2,3-BD with a high concentration of 111.0 g/L, the fermentation process needed addition of 60.0 g/L YE. *B. licheniformis* DSM8785 also produced 2,3-BD with a rather high concentration (144.7 g/L), but the productivity of the strain was unsatisfactory. *B. licheniformis* 10-1-A produced 2,3-BD with a yield of 94% and an average productivity of 2.4 g/L·h. As shown in Table 8, the yield and productivity of strain 10-1-A are both the new record of 2,3-BD production by GRAS strain.

**Table 8:** Comparison of the 2,3-BD production using different microorganisms

**Strain**	**Temperature**	**Concentration**	**Productivity**	**Yield**	**Reference**
	**(°C)**	**(g/L)**	**(g/L·h)**	**(%)**	
*B. amyloliquefaciens*	37	92.3	0.96	84	12
*B. subtilis*	37	6.1	0.4	67	13
*S. cerevisia*	ND	2.3	0.03	22.6	14
*P. polymyxa*	30	111	2.06	ND	11
*Geobacillus* sp.	55	14.5	0.30	69	17
*B. licheniformis*	30	144.7	1.14	80	10
*B. licheniformis*	50	12.1	1.01	90	15
*S. marcescens*	30	152	2.67	82	9
*K. oxytoca*	37	130	1.64	96	32
*K. pneumoniae*	37	150	4.21	86	8
*B. licheniformis*	50	115	2.4	94	This study

The high yield of strain 10-1-A would not only decrease the substrate cost but also make the downstream processing much easy and inexpensive to perform. The high productivity of strain 10-1-A might be a result of the high metabolic rate at its relatively high optimal fermentation temperature. As shown in Table 8, as a novel GRAS strain, *B. licheniformis* 10-1-A produced 2,3-BD with a productivity that compares favourably with those of risk group 2 species. Thus, although pathogenic *K. pneumonia*, *K. oxytoca, Enterobacter cloacae* and *S. marcescens* produced 2,3-BD with higher concentrations [8,9,32,33], the thermophilic *B. licheniformis* strain is still a very promising alternative for the large scale production of 2,3-BD.

All of the isomers of 2,3-BD including (2*R*,3*R*)-2,3-BD, (2*S*,3*S*)-2,3-BD and *meso*-2,3-BD are important potential chemical intermediates. Different microorganisms produce different stereoisomers of 2,3-BD [18]. Thus, isomers formation mechanisms and the key enzymes in different 2,3-BD producers such as *K. pneumonia, P. polymyxa* and *S. marcescens* have been studied to make the fermentation process more clearly and efficiently [18,19,23]. However, the 2,3-BD formation mechanism in *B. licheniformis*, a very promising 2,3-BD producer, has never been studied until now.

Based on the genome sequence of thermophilic *B. licheniformis* strain 10-1-A [27], the key enzymes involved in 2,3-BD formation were analyzed. A complete *alsSD* operon was found in the genome of the strain. The *alsS* gene may encode an α-acetolactate synthase that catalyzes the conversion of pyruvate to α-acetolactate. The *alsD* gene may encode an α-acetolactate decarboxylase that catalyzes the conversion of α-acetolactate to (3*R*)-AC. The *alsR* gene, which is located upstream of the *alsSD* operon, may encode the transcriptional regulator AlsR. The protein sequences of these genes exhibit high identities with those of *K. pneumonia, B. subtilis*, *E. cloacae* and *S. marcescens* (Additional file 2: Figure S2). Since the reaction catalyzed by α-acetolactate decarboxylase is stereospecific and only leads to the formation of the (3*R*)-AC [34], stereospecificity of the BDH has been accepted as a key factor for 2,3-BD isomers formation in *K. pneumonia, P. polymyxa*, *E. cloacae* and *S. marcescens*. Thus, the putative BDH in strain 10-1-A was studied detailedly in this work.

Strains of *Bacillus* sp., such as *P. polymyxa*, produced (2*R*,3*R*)-2,3-BD as the major product, (2*R*,3*R*)-BDH was found to be responsible for the production of (2*R*,3*R*)-2,3-BD in *P. polymyxa*[19]. Some pathogenic strains including *K. pneumonia, E. cloacae* and *S. marcescens* produce *meso*-2,3-BD as the major product. The *meso*-BDHs are responsible for production of *meso*-2,3-BD in those strains [18,21,23]. As shown in Figure 6, different from all the reported 2,3-BD producing strains mentioned above, strain 10-1-A produced a mixture of (2*R*,3*R*)-2,3-BD and *meso*-2,3-BD with a ratio of nearly 1:1. As (3*R*)-AC would be the major source of (2*R*,3*R*)-2,3-BD and *meso*-2,3-BD, a *meso*-BDH (catalyzing (3*R*)-AC into *meso*-2,3-BD) and a (2*R*,3*R*)-BDH (catalyzing (3*R*)-AC into (2*R*,3*R*)-2,3-BD) would be co-present in strain 10-1-A.

After analysis of the genome sequence, a *bdh* gene encoding a *meso*-BDH of SDR family was found in the genome of strain 10-1-A. Its protein sequence exhibits 67.7% identity with that of *K. pneumonia* IAM1063 (Additional file 1: Figure S1). This enzyme was purified and its stereospecificity was studied. *meso*-2,3-BD was found to be the major product from racemic AC. This property is similar to the *meso*-BDH in *S. marcescens* which is key enzyme for *meso*-2,3-BD production [18].

No gene exhibiting high identity with (2*R*,3*R*)-BDHs of *P. polymyxa* ATCC12321, *B. subtilis* 168, and *S. cerevisiae*, was found in the genome sequence of strain 10-1-A. Glycerol is a vicinal diol with a similar structure to 2,3-BD except the first hydroxyl group. It was reported that GDH in *O. angusta* had the ability to catalyze interconversion between (3*R*)-AC and (2*R*,3*R*)-2,3-BD and this enzyme showed greater activity towards (2*R*,3*R*)-2,3-BD than towards glycerol [25]. This indicated that GDH might play a role in 2,3-BD formation. Thus, the *gdh* gene encoding a GDH of MDR family in strain 10-1-A was also cloned and expressed in *E. coli* (Additional file 1: Figure S1). Although the protein sequence of GDH in strain 10-1-A is only 11.8% identity with that of *O. angusta* (BAD32688.1), the enzyme could also catalyze the production of (2*R*,3*R*)-2,3-BD from racemic AC. Thus the *meso*-BDH and GDH would be the enzymes for the *meso*-2,3-BD and (2*R*,3*R*)-2,3-BD production in strain 10-1-A, respectively.

## Conclusions

In the present work, a GRAS thermophilic strain, *B. licheniformis* 10-1-A, was isolated for 2,3-BD production at 50°C. This strain produced *meso*-2,3-BD and (2*R*,3*R*)-2,3-BD as the major product. The key enzymes for *meso*-2,3-BD and (2*R*,3*R*)-2,3-BD production were studied. In a 5-L fed-batch fermentation of *B. licheniformis* 10-1-A, 115.7 g/L of 2,3-BD was produced in 48 h. The fermentation process exhibited rather high yield and productivity of 2,3-BD, indicating that *B. licheniformis* 10-1-A might be a promising 2,3-BD producer.

## Materials and methods

### Materials

(2*R*,3*R*)-2,3-BD (98.0%), (2*S*,3*S*)-2,3-BD (99.0%), and *meso*-2,3-BD (98.0%) were purchased from ACROS (The Kingdom of Belgium). CSLP was purchased from Shanghai Xiwang Starch Sugar CO., Ltd (Shanghai, China). All other chemicals were of analytical grade and commercially available.

### Isolation of bacteria for 2,3-BD production

Soil samples were collected from various areas, including farmland, gardens and orchard lands. Approximately 5 g of each was enriched in 100 mL of MRS medium (50 g/L of glucose) and incubated at 50°C with agitation for 12 h. An aliquot of the broth was plated on MRS agar medium (18 g/L of agar) and cultivated at 50°C for 12 h. Based on colony type and size, representative colonies were selected and maintained in 100 mL MRS medium. After 8 h of incubation at 50°C, the strains that are Voges-Proskauer-positive were selected in the medium containing (g/L): glucose 120, YE 5, peptone 10, beef extract 10, triammonium citrate 2, sodium acetate 4, K_2_HPO_4_·3H_2_O 2, MgSO_4_·7H_2_O 0.2 at pH 7.0. After 30 h of incubation at 50°C, the strain produced the highest concentration of 2,3-BD, designated as 10-1-A, was selected for further analysis.

### Microorganism and culture conditions

Strain 10-1-A was maintained on MRS agar slants. The slants were incubated at 50°C and the fully grown slants were stored at 4°C. The medium for inoculation contained (g/L): glucose 50.0, peptone 10.0, YE 5, NaCl 10.0. The seed culture was prepared as follows: a loop of cells from the fully grown slant was inoculated into 100 mL of the above medium in 500-mL Erlenmeyer flasks and incubated at 50°C for 12 h with agitation. Then the seed culture was inoculated into Erlenmeyer flasks or bioreactors (inoculum volume at 5%) for 2,3-BD production.

### Genetic and physiological characterization

The partial 16S rDNA was amplified by Polymerase Chain Reaction (PCR) from the genome of strain 10-1-A using primers 27 F and 1492R. The primers were as follows: 27 F, 5’-GAGAGTTTGATCCTGGCTCAG-3’ and 1492R, 5’-ACGGCTACCTTGTTACGACTT-3’. The PCR program consisted of 30 repetitive cycles with a strand separation step at 94°C for 30 s, an annealing step at 55°C for 30 s and an elongation step at 72°C for 30 s. The biochemical and physiological characteristics of strain 10-1-A, such as starch hydrolysis, the VP reaction, growth in 6.5% NaCl and at 55°C, were determined step by step according to Bergey’s manual of Determinative Bacteriology [35].

### Optimization of fermentation temperature and initial glucose concentration

The optimal fermentation temperature of strain 10-1-A was tested using the medium contained (g/L): glucose 90, peptone 10, YE 5, beef extract 10.0, triammonium citrate 2.0, sodium acetate 4.0, K_2_HPO_4_·3H_2_O 2.0, MgSO_4_·7H_2_O 0.2. Fermentations were conducted in 500-mL Erlenmeyer flasks containing 100 mL of medium with shaking at 180 rpm on a rotary shaker. After 24 h of fermentation at 37°C, 42°C, 50°C, 55°C and 60°C, respectively, samples were taken and subjected for assay of 2,3-BD.

The following medium that contained (g/L): peptone 10.0, YE 5.0, beef extract 10.0, triammonium citrate 2.0, sodium acetate 4.0, K_2_HPO_4_·3H_2_O 2.0, MgSO_4_·7H_2_O 0.2 was used to study the optimal initial glucose concentration. The experiments were conducted in 100 mL of each medium with initial glucose concentrations at 64.0, 91.0, 125.0, 154.0 and 180.0 g/L and shaken at 180 rpm on a rotary shaker. Samples were taken at 9, 13, 17 and 22 h and the concentrations of residual glucose and produced 2,3-BD were determined.

### Selection of organic nitrogen source

For selection of organic nitrogen source, the following medium that contained (g/L): glucose 90, triammonium citrate 2, sodium acetate 4, K_2_HPO_4_·3H_2_O 2, MgSO_4_·7H_2_O 0.2, was used. The organic nitrogens source used were as follows: 1, 0.5% YE, 1% peptone and 1% beef extract; 2, 1% CSLP; 3, 1% CSLP and 0.5% YE; 4, 1% CSLP and 0.5% peptone; 5, 1% CSLP and 0.5% beef extract. The experiments were conducted in 500-mL Erlenmeyer flasks containing 100 mL of each medium with shaking at 180 rpm on a rotary shaker at 50°C. Samples were taken after 24 h and the concentrations of 2,3-BD were determined.

### Statistical optimization of fermentation medium

Plackett–Burman design, an efficient technique for medium component optimization, was used to pick factors that significantly influenced 2,3-BD production, and insignificant factors that should be eliminated to obtain a smaller, more manageable set of factors. A 12-run Plackett–Burman design was used to screen six factors. The experimental responses were analyzed by the method of least squares to fit the following first-order model:

(3) $$\begin{array}{ll} \;Y= & \;\beta_{0}+\beta_{1}X_{1}+\beta_{2}X_{2}+\beta_{3}X_{3}+\beta_{4}X_{4}+\beta_{5}X_{5}\\ & \;+\beta_{6}X_{6} \end{array}$$

where *Y* was the predicted response (2,3-BD concentration), *β*_0_, *β*_1_, *β*_2_, *β*_3_, *β*_4_, *β*_5_, and *β*_6_ were the regression coefficients, and *X*_1_, *X*_2_, *X*_3_, *X*_4_, *X*_5_, and *X*_6_ were the coded levels of the independent variables.

Based on the first-order model equation obtained by the Plackett–Burman design, a series of trials were performed in the direction of the steepest ascent. To fit the empiric second-order polynomial model, a CCD with five coded levels was performed. The quadratic model for predicting the optimal point was expressed according to the following equation:

(4) $$\begin{array}{ll} \;Y=\; & \;\beta_{0}+\beta_{1}X_{1}+\beta_{2}X_{2}+\beta_{3}X_{3}+\beta_{11}X_{1}{}^{2}\\ & \;+\beta_{22}X_{2}{}^{2}+\beta_{33}X_{3}{}^{2}+\beta_{12}X_{1}X_{2}+\beta_{23}X_{2}X_{3}\\ & \;+\beta_{13}X_{1}X_{3} \end{array}$$

where *Y* was predicted response (2,3-BD concentration); *β*_0_ was intercept; *β*_1_, *β*_2_, and *β*_3_ were linear coefficients, *β*_11_, *β*_22_, and *β*_33_ were squared coefficients; *β*_12_, *β*_23_, and *β*_13_ were interaction coefficients, and *X*_1_, *X*_2_ and *X*_3_ were the coded levels of the independent variables. SAS package (version 9.0, SAS Institute, Cary, USA) was used for all the statistical analysis and the response surface plotting. Besides the studied components, the fermentation media of all the statistical optimization experiments contained about 90 g/L of glucose.

All of the experiments mentioned above were conducted in 500-mL Erlenmeyer flasks containing 100 mL of each medium with shaking at 180 rpm on a rotary shaker at 50°C. Samples were taken at 24 h and the concentrations of 2,3-BD were determined.

### Batch and fed-batch fermentations

Batch and fed-bacth fermentations were conducted in a 1-L bioreactor (Infors AG, Bottmingen, Switzerland) with 0.8 L initial medium or 5-L bioreactor (BIOSTAT B, B. Braun Biotech International GmbH, Germany) with 4 L initial medium, respectively. The seed culture prepared as mentioned above was inoculated (5%, v/v) into the optimized fermentation medium. The cultivation was carried out at 50°C, stirring at 400 rpm, and airflow at 1.0 vvm. The pH was maintained by automatic addition of 6 M NaOH and 6 M acetic acid using a program-controlled peristaltic pump. Samples were collected periodically to determine the biomass, concentrations of glucose, 2,3-BD and by-products.

### Expression and purification of *meso*-BDH and GDH

The primers, B1 and B2, were used to obtain the *bdh* gene from strain 10-1-A While G1and G2 were used to obtain the *gdh* gene. B1 with an *EcoR*I restriction site insertion and B2 with a *Sal*I restriction site insertion were as follows: B1, 5′CGAATTCAATGAGTAAAGTATCTGGAAA3′ and B2, 5′GTCGACTTAATTAAATACCATTCCGC3′. G1 with a *BamH*I restriction site insertion and G2 with a *Hind*III restriction site insertion were as follows: G1, 5′TGGATCCAATGTCAAAATCAGTAAAATCAG3′ and G2, 5′AAGCTTTTAATCGTGATAAGATTCTGC3′. The genes were amplified by Polymerase Chain Reaction from the genome of strain 10-1-A using B1, B2, G1, and G2. The PCR program consisted of 30 repetitive cycles with a strand separation step at 94°C for 30 s, an annealing step at 60°C for 30 s and an elongation step for 30 s at 72°C, and then the gene was ligated to the pEASY-Blunt vector (Transgen, China). The constructed vector was named pEASY-Blunt-*bdh* and sequenced (Sangon, Shanghai, China). To construct the *bdh* expression vector under the control of promoter T7, pEASY-Blunt-*bdh* was digested with *EcoR*Ι and *Sal*I, and the gel-purified *bdh* fragment was ligated to the pETDuet-1 vector digested with the same restriction enzymes. The resulting plasmid was designated pETDuet-*bdh*. The pETDuet-*gdh* plasmid was obtained by the same way.

Recombinant *E. coli* cells were grown at 37°C on a rotary shaker (180 rpm) in LB medium containing ampicillin (100 μg/mL) to an OD_620nm_ of 0.6. Expression of the recombinant gene was induced by adding 1 mM IPTG at 16°C to avoid the formation of inactive inclusion bodies for about 10 h. After induction, the cells were harvested by centrifugation at 6,000 × *g* for 5 min at 4°C and then washed twice with 0.85% NaCl. The harvested cells were resuspended in binding buffer, containing 20 mM potassium phosphate, 500 mM NaCl, 20 mM imidazole, 0.1 mM PMSF, and 1 mM dithiothreitol at pH 7.4, and were disrupted by sonication in an ice bath. The homogenate was centrifuged at 15,000 × *g* for 30 min, and the supernatant (crude extract) was recovered. Crude extract was loaded on a 5 mL HisTrap HP column (GE Healthcare, USA), which had been equilibrated with 25 mL binding buffer. The column was washed with binding buffer, and proteins were then eluted with 10%, 60%, and 100% elution buffer, containing 20 mM potassium phosphate buffer, 500 mM NaCl, and 500 mM imidazole (pH 7.4). The 60% fraction was collected, concentrated, and desalted in an Amicon Ultra-15 Centrifugal Filter Units (10 kDa) with 50 mM Tris–HCl (pH 7.4). The expressed and purified enzymes were determined by SDS-PAGE.

### Analytical methods

Samples were withdrawn periodically and centrifuged at 12,000 × *g* for 10 min. The concentration of glucose was measured enzymatically by a bio-analyzer (SBA-40D, Shandong Academy of Sciences, China) after diluting to an appropriate concentration. The analytical methods of the concentration and the stereoisomers of 2,3-BD were the same as that described in Ma et al. [8], and Xiao et al. [24]. The concentrations of by-products including acetic acid, formic acid, lactic acid and ethanol, were measured by high-performance liquid chromatography (HPLC, Agilent 1100 series, Hewlett-Packard), equipped with a Bio-Rad Aminex HPX-87H column (300 × 7.8 mm) and a refractive index detector as described in Li et al. [21].

## Competing interests

The authors declare that they have no competing interests.

## Authors’ contributions

LL, CM and PX designed the study. LL, LZ, KL YW, and BH executed the experimental work. LL, YW, and CG analysed the data. CM, CG and PX contributed reagents and materials. LL, CG, CM and PX wrote and revised the manuscript. All authors read and approved the final manuscript.

## Supplementary Material

Additional file 1: Figure S1 Multiple sequence alignment and phylogenetic tree analysis of the enzymes for 2,3-BD production in strain 10-1-A and the reported 2,3-butanediol dehydrogenases (BDHs). Click here for file

Additional file 2: Figure S2 The *alsSD* operon in different strains. Click here for file
