# Supplementary material for: A newly isolated Bacillus licheniformis strain thermophilically produces 2,3-butanediol, a platform and fuel bio-chemical
Source: Biotechnol Biofuels. 2013 Aug 28;6:123. doi: 10.1186/1754-6834-6-123 (PMC3766113; doi:10.1186/1754-6834-6-123)
Supplement: Additional file 1: Figure S1 — Multiple sequence alignment and phylogenetic tree analysis of the enzymes for 2,3-BD production in strain 10-1-A and the reported 2,3-butanediol dehydrogenases (BDHs). [file 1754-6834-6-123-S1.pdf]

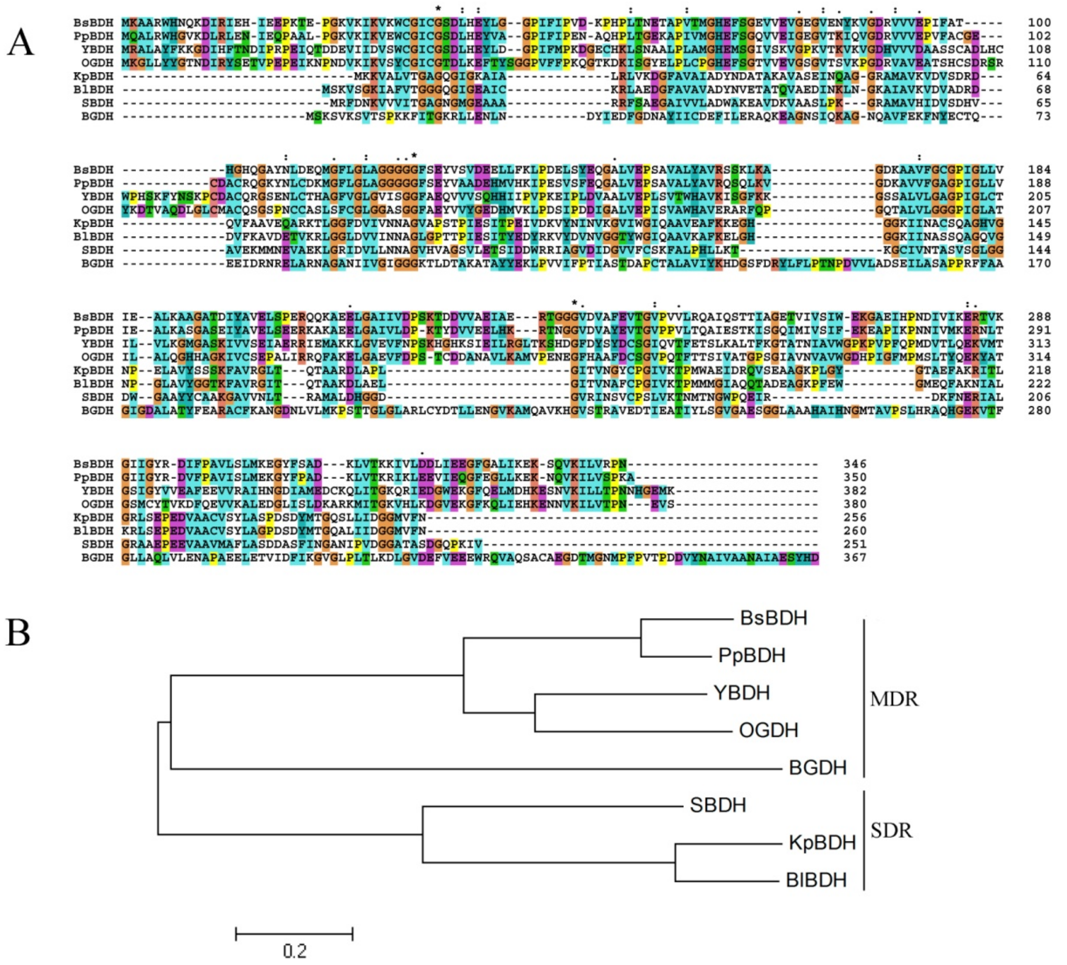

Figure S1 Multiple sequence alignment and phylogenetic tree analysis of the enzymes for 2,3-BD production in strain 10-1-A and the reported 2,3-butanediol dehydrogenases (BDHs).

A, Multiple sequence alignment of BDHs; B, phylogenetic tree analysis of the BDHs. PpBDH, the (2*R*,3*R*)-BDH in *Paenibacillus polymyxa* (GenBank: YP_001487573); YBDH, the (2*R*,3*R*)-BDH in *Saccharomyces cerevisiae* (GenBank: AAC04974.1); BsBDH, the (2*R*,3*R*)-BDH in *B. subtilis* (GenBank: NP_388505.1); SBDH, *meso*-BDH in *S. marcescens* BDH (GenBank: AFH00999); KpBDH, *meso*-BDH in *K. pneumoniae* IAM 1063 (GenBank: YP_002919837.1); OGDH, the glycerol dehydrogenase (GDH) in *Odostomia angusta* (GenBank: BAD32688.1); BlBDH, *meso*-BDH in strain 10-1-A (GenBank: KF250429); BGDH, GDH in strain 10-1-A (GenBank: KF250430).
