# Supplementary material for: A newly isolated Bacillus licheniformis strain thermophilically produces 2,3-butanediol, a platform and fuel bio-chemical
Source: Biotechnol Biofuels. 2013 Aug 28;6:123. doi: 10.1186/1754-6834-6-123 (PMC3766113; doi:10.1186/1754-6834-6-123)
Supplement: Additional file 2: Figure S2 — The alsSD operon in different strains. [file 1754-6834-6-123-S2.pdf]

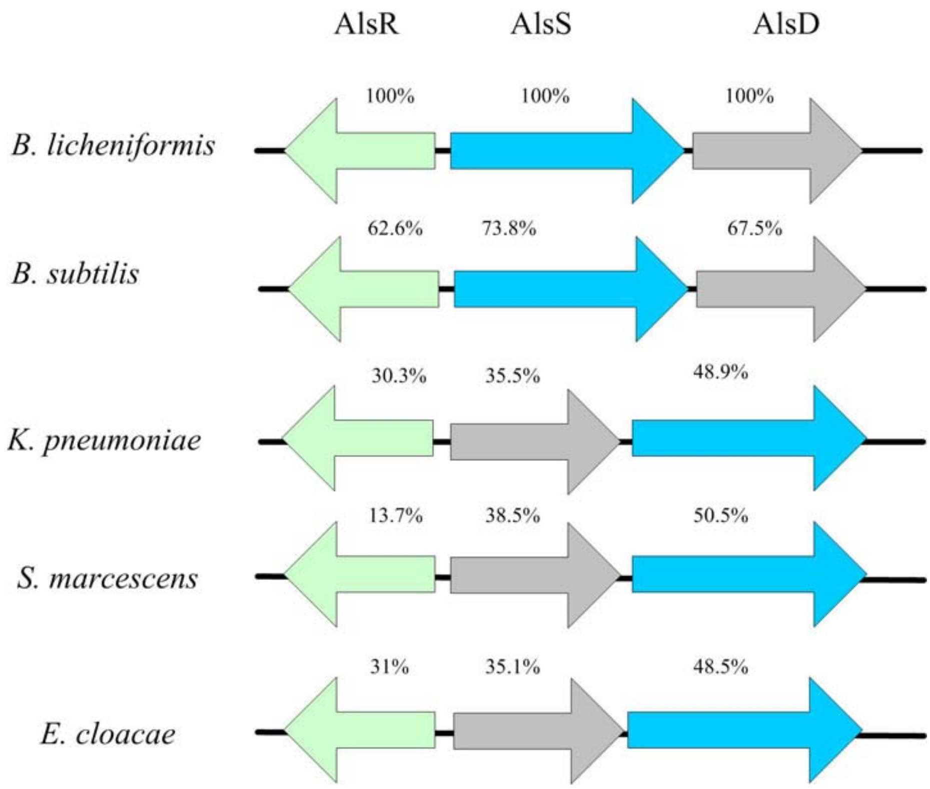

Figure S2 The *alsSD* operon in different strains.

The *alsSD* opreons include that of *Bacillus licheniformis* 10-1-A (AJLV00000000.1), *B. subtilis* 168 (NC_000964.3); *Serratia marcescens* MG1 (JF519735, JF519736, JF519737); *Klebsiella pneumoniae* NTUH-K2044 (NC_012731.1); *Enterobacter cloacae* subsp. *dissolvens* SDM (CP003678.1). The numbers represent the identities of the protein sequences in different strains with that of strain 10-1-A.
